# Supplementary material for: Development of a computer-aided design software for the quantitative evaluation of aesthetic damage
Source: PLoS One. 2019 Dec 18;14(12):e0226322. doi: 10.1371/journal.pone.0226322 (PMC6919621; doi:10.1371/journal.pone.0226322)
Supplement: S1 Code site — 1. (ZIP) [file pone.0226322.s001.zip › [EESC_jr][Projeto]Relatorio_07_21/reports/templates_zip/gentelella-master/vendors/Flot/examples/basic-usage/index.html]

Flot Examples: Basic Usage


## Basic Usage

You don't have to do much to get an attractive plot. Create a placeholder, make sure it has dimensions (so Flot knows at what size to draw the plot), then call the plot function with your data.

The axes are automatically scaled.

Copyright © 2007 - 2014 IOLA and Ole Laursen
